# The neural dynamics of loss aversion

**DOI:** 10.1162/imag_a_00047

**Published:** 2023-12-18

**Authors:** Shaozhi Nie, Muzhi Wang, Jian Li, Huan Luo, Hang Zhang

**Affiliations:** School of Psychological and Cognitive Sciences and Beijing Key Laboratory of Behavior and Mental Health, Peking University, Beijing, China; Department of Psychology, University of Minnesota Twin Cities, Minneapolis, Minnesota, United States; PKU-IDG/McGovern Institute for Brain Research, Peking University, Beijing, China; Key Laboratory of Machine Perception, Ministry of Education, Peking University, Beijing, China; Peking-Tsinghua Center for Life Sciences, Peking University, Beijing, China; Chinese Institute for Brain Research, Beijing, China

**Keywords:** decision under risk, loss aversion, MEG, Prospect Theory

## Abstract

In human decision-making under risk, loss is typically valued more than the same amount of gain, a behavioral phenomenon known as loss aversion, which suggests that gain and loss are evaluated differently in the brain. Most previous neuroimaging studies focused on the brain regions that show differential responses to losses relative to gains. What is still largely unknown is how the neural processing of gain and loss may unfold in time and drives loss aversion. Here, we designed a gambling task ideal for investigating the temporal course of the valuation process and used magnetoencephalography (MEG) to track human participants’ brain activities for valuating gain and loss. Computational modeling of participants’ behaviors implies that the gain and loss presented simultaneously can compete for cognitive resources, during which loss signals dominate the valuation process, resulting in loss aversion. Indeed, time-resolved MEG analysis reveals that the evaluation process of loss terminated later for participants with higher loss aversion than those with lower loss aversion, though the gain valuation had similar temporal courses for different participants. These results suggest that the origin of loss aversion may lie in the neural dynamics of loss processing

## 1 Introduction

Loss aversion—that is, “losses loom larger than gains”—is a key assumption of Kahneman and Tversky’s (1979) Prospect Theory, which provides a parsimonious explanation for a range of seemingly irrational behaviors in human decision under risk. As a straightforward experimental proof, people tend to reject gambles with equal possibilities to gain or lose money unless the potential gain is much higher than loss, which is observed in both laboratory (Tom et al., 2007) and large-scale web experiments (Brown et al., 2014). Real-world examples include house sellers’ avoidance of realizing nominal loss (Genesove & Mayer, 2001), cabdrivers’ stickiness to daily earning targets (Camerer et al., 1997), and one’s faster drop in happiness for income drops below social reference level (Vendrik & Woltjer, 2007). Many economic effects such as the equity premium puzzle (Benartzi & Thaler, 1995), the status quo bias, and the endowment effect (Kahneman et al., 1991) can also be explained in the framework of loss aversion.

Why are people loss averse in decisions under risk? Or, given that not all individuals are risk averse, why are some individuals more loss averse than others? Though part of the loss aversion effects may be explained by biases in response rather than in valuation (Gal, 2006; Zhao et al., 2020), other studies agree with a valuation difference between gain and loss as Kahneman and Tversky originally assumed (McGraw et al., 2010; Rick, 2011; Sokol-Hessner & Rutledge, 2019). As uncovered by functional MRI (fMRI) studies, many brain regions associated with valuation (ventral striatum and ventral medial prefrontal cortex) or negative emotions (amygdala, posterior insula, and parietal operculum) respond stronger to loss than to gain (Canessa et al., 2013; Sokol-Hessner et al., 2013; Tom et al., 2007). Their sensitivity or relative sensitivity to loss is correlated with the extent of loss aversion in individuals’ decision behavior (Canessa et al., 2013; Tom et al., 2007). The resting-state activity in some of these areas also correlates with individuals’ behavioral loss aversion (Canessa et al., 2017).

What is largely missing in this picture, due to the limited temporal resolution of fMRI, is the temporal dynamics in evaluating gains and losses. A few fMRI studies reported the time courses of value representation (e.g., Breiter et al., 2001). More precise measurements such as Electroencephalogram (EEG) and magnetoencephalography (MEG) could be used to track the decision process on the scale of milliseconds. Though some EEG studies found correlations between individuals’ loss aversion and classic neural signatures such as conflict-related event-related potential (ERP) (Heeren et al., 2016) and response-related ERP (Zeng et al., 2019), they informed us little about the representation of values. A few studies examined value representation (Hunt et al., 2012; Pornpattananangkul et al., 2019), but their focus was on the value integration upon choice selection, effectively assuming a symmetry between gains and losses. As we know from fMRI studies (Canessa et al., 2013; Tom et al., 2007), the asymmetry in gain-loss representation, especially in the same brain areas, is essential for the occurrence of loss aversion. We are interested in uncovering such gain-loss asymmetry in temporal dynamics.

From the theoretical perspective, understanding the temporal dynamics of gain and loss valuation can help to distinguish between two alternative hypotheses concerning the neural basis of loss aversion. One is the static sensitivity hypothesis, a straightforward interpretation of the domain-specific utility functions assumed in the Prospect Theory (Kahneman & Tversky, 1979), which hypothesizes that neural encoding sensitivity or response strength is different for gains and losses. Evidence for this hypothesis mainly comes from the above-mentioned fMRI studies (Canessa et al., 2013; Li et al., 2019; Sokol-Hessner et al., 2013; Tom et al., 2007). However, this line of evidence is also compatible with an alternative dynamic context hypothesis which states that valuation results from the cognitive competition processes due to the limitation of cognitive resource. Similar to the key proposition in the attentional drift diffusion model (Krajbich et al., 2010), this hypothesis assumes that gains and losses compete for limited cognitive resources, with the over-valuation of loss arising as the consequence of a more intense processing of losses than gains. The dynamic context hypothesis is consistent with the findings that loss aversion can be attenuated by attention manipulation (Sokol-Hessner et al., 2009; Thaler et al., 1997) and may even disappear when gain and loss are presented in isolation (McGraw et al., 2010). The static sensitivity and dynamic context hypotheses differ in their predictions about the neural dynamics of gain and loss valuation: The former predicts greater encoding strength for loss than for gain regardless of time, while the latter only predicts an overall bias towards loss, which may result from differences in either encoding strength or time course (Clay et al., 2017; Sheng et al., 2020; Zhao et al., 2020).

**Fig. 1.**
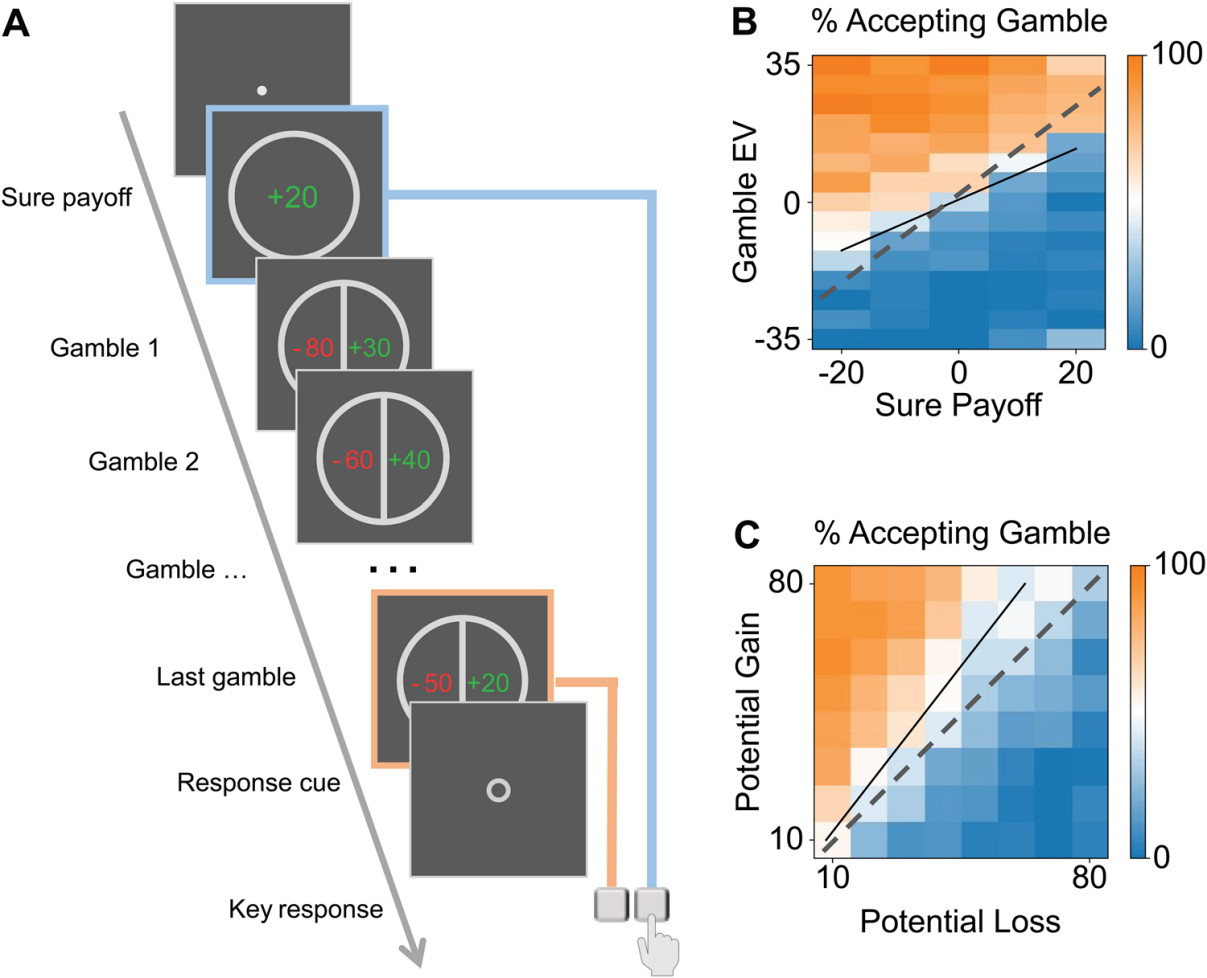
Task and choice summary. (A) Stimuli sequence of a trial. Each trial started with a single number representing a sure payoff. Then, a sequence of pairs of a positive number and a negative number was shown, each representing a gamble with 50%–50% probabilities for gain and loss. Participants were asked to choose between the sure payoff and the last gamble. No feedback was given. (B) Probability of accepting the gamble as a function of its expected value (EV) and the sure payoff. (C) Probability of accepting the gamble as a function of its potential gain and loss, averaged across sure payoff levels, shown in a format similar to previous loss aversion studies (Canessa et al., 2013; Tom et al., 2007). The black lines represent the estimated lines of a 50% probability of accepting the gamble, and dashed lines represent those for a theoretical loss-neutral participant.

In the present study, we investigated the neural dynamics of gain and loss valuation and used MEG to track human participants’ brain activities during an adapted version of the classic gambling task. On each trial (Fig. 1A), participants saw a sure payoff followed by a sequence of gambles, each with independently varied gains and losses. Participants were required to choose between the sure payoff and the gamble appeared last in the trial. We performed a time-resolved decoding analysis to the MEG signals following each gamble and found that the neural valuation of gain and loss lasted for approximately 2 s for each gamble, far after the offset of the gamble and the onset of the next gamble. To reveal the behavioral relevance of these neural signals, we divided participants into two groups with higher and lower loss aversion (λ) according to their choices. The two groups had similar encoding strength for gain, but the high-λ group had a stronger encoding of loss than the low-λ group in the later period of the valuation process. This finding and computational modeling of participants’ choice behaviors provide converging evidence for the dynamic context hypothesis.

## 2 Methods

### 2.1 Participants

Twenty-six university students (15 males and 11 females; mean age of 21.8 years, SD = 2.3 years) from Peking University participated in the experiment. All participants had normal or corrected-to-normal vision. Participants received a baseline payoff of 120 CNY for 1.5 hours of time and a performance-based bonus ranging from 0 to 160 CNY. The study had been approved by the Institutional Review Board of School of Psychological and Cognitive Sciences at Peking University and all participants provided informed consent before the experiment.

### 2.2 Experiment

#### 2.2.1 Apparatus

Participants were seated approximately 80 cm in front of a projection screen (Panasonic PT-DS12KE: 49.6 × 37.2 cm, 1024 × 768 pixels, 60-Hz refresh rate) inside the magnetically shielded room. Their behavioral responses were recorded by an MEG-compatible response pad and their brain activities by a 306-channel MEG system (Elekta-Neuromag, 102 magnetometers and 102 pairs of orthogonal planar gradiometers).

#### 2.2.2 Task and procedure

We used a gambling task adapted from previous fMRI studies on loss aversion (Canessa et al., 2013; Tom et al., 2007). In previous studies, participants were asked to choose whether they prefer to accept a gamble consisting of equal probabilities of yielding monetary gain and loss, or prefer to receive nothing. Our task differed from previous studies in the following two aspects. First, we allowed the sure payoff to be non-zero and to vary across trials and this variation enabled us to tell apart different hypotheses on the gain and loss valuation. Second, we presented a series of gambles instead of a single gamble in each trial, and required participants to decide only for the last gamble. Such manipulation allowed us to acquire MEG data less contaminated by the response-related brain activities.

On each trial (Fig. 1), after a white fixation circle was presented for 500 ms, a sure payoff was presented for 2500 ms, followed by a blank screen of 250 ms and then a sequence of gambles with equal probabilities of gain and loss realization. Each gamble was shown for 1000 ms, with a 250 ms interstimulus interval (ISI) between adjacent gambles. The values of gains and losses in the sure payoff or gambles were represented by numbers in different colors (red or green), with the color code counterbalanced across participants. After the last gamble, a circle prompted participants to choose whether they preferred the sure payoff or the last gamble.

To encourage participants to encode each gamble, 4 out of 5 of the trials were regular trials with 7 gambles but 1 out of 5 of the trials were catch trials whose sequence length followed a truncated and discretized exponential distribution (1–12 gambles, mean 7 gambles), so that participants were unable to know in advance whether a gamble was the last. Only the regular trials were submitted to behavioral and MEG analyses.

Participants were given the following task instructions (in Chinese): “A sure payoff will be followed by several gambles. When an ‘o’ appears on the screen, press button to choose whether you accept the current gamble. Press 1 to ignore the gamble and accept the sure payoff; Press 2 to accept the gamble and ignore the sure payoff. ‘The current gamble’ refers to the last gamble before ‘o’. The ‘o’ can appear at any time. Pay attention to every gamble so that you will not miss it.”

Following the common practice of previous gambling studies (Canessa et al., 2013; De martino et al., 2010; Tom et al., 2007), we withheld feedback in the experiment. At the end of the experiment, one trial was randomly chosen to be realized. Each participant was endowed 80 CNY at the beginning of the experiment, so that they were in a frame of possibly losing money but would not result in negative bonus even in the worst case (i.e., −80 CNY).

#### 2.2.3 Design of gambles and sure payoffs

We set the ranges of gain and loss to be symmetric and varied the two values independently. For each participant, there were 8 levels of monetary gains ranging from 10 to 80 CNY in increments of 10, and similarly 8 levels of losses from –10 to –80 CNY, thus forming 64 gain-loss pairs. Each pair was repeated 3 times, with the sure payoff randomly drawn from –20, –10, 0, +10, +20 in ratios of 1:2:3:2:1. For each participant we hereby generated 192 trials (including the catch trials) with a total of 1228 to 1283 gambles (mean 1260 gambles), and presented all trials and the gambles in each trial in random order. Participants were required to rest for at least 1 minute after every 20 trials.

### 2.3 Data analysis

#### 2.3.1 Behavioral modeling

We only analyzed trials with 7 gambles and excluded trials with response time longer than 2 s, or response before the response cue (17 trials for 13 participants). Analysis including catch trials showed similar results (Figs. S6 & S7). To be consistent with later MEG analysis, we excluded trials with invalid MEG recordings (1 trial from each of two participants, see MEG Acquisition and Preprocessing for details). For each participant, 152 to 158 trials were included for the MEG data analysis (Mean 155 trials).

**Fig. 2.**
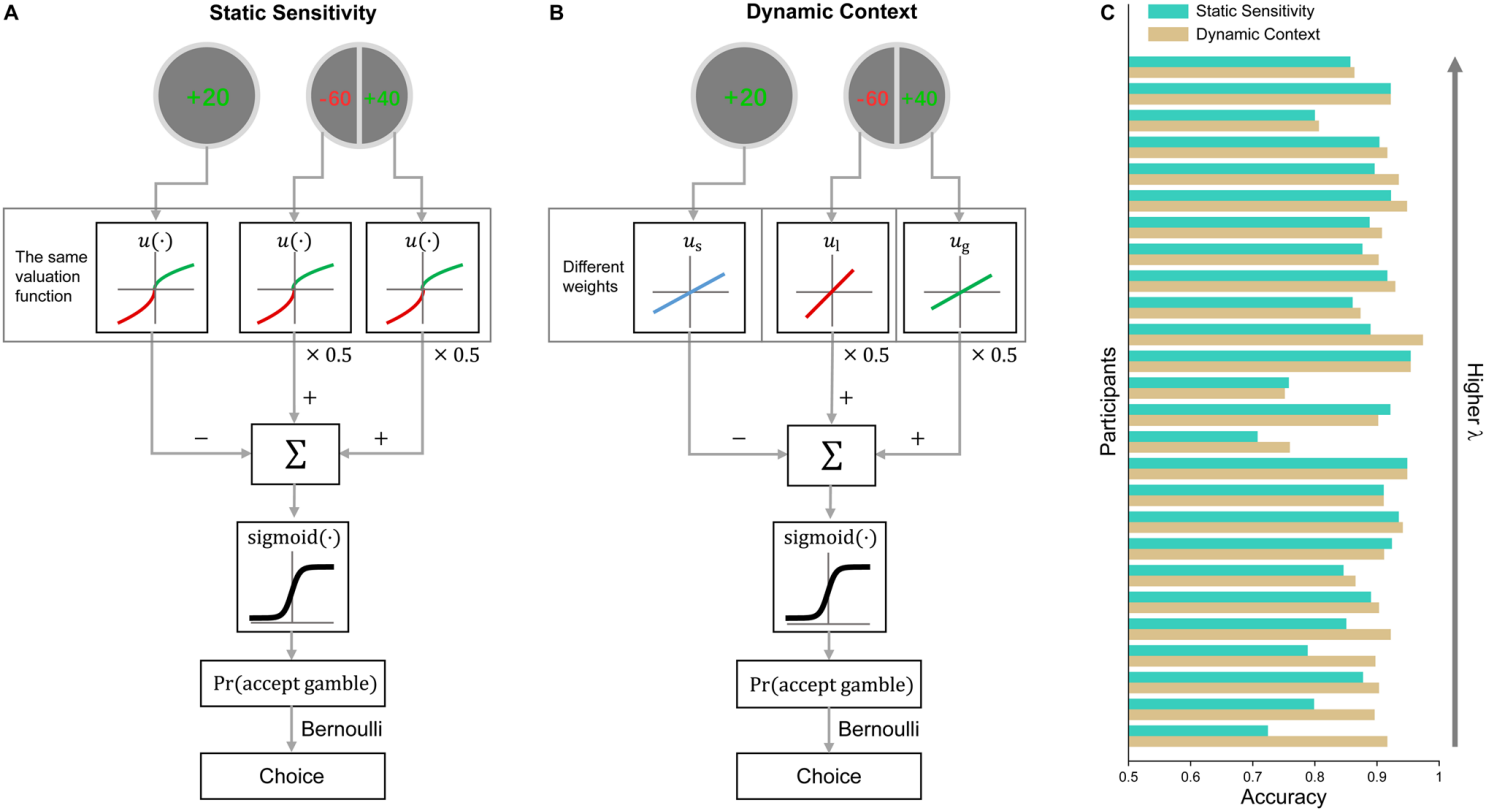
Behavioral models and model comparison results. (A) The static sensitivity model assumes different utility functions for gains and losses, no matter whether they are in gambles or sure payoffs. (B) The dynamic context model assumes different utility functions for gains and losses in gambles but the same utility function for those as sure payoffs. The decision process of the two models is otherwise identical. (C) Accuracy of the two models for each participant.

We constructed two alternative models for participants’ behavioral choices (see Fig. 2 for illustration of assumptions). The static sensitivity model is based on the Prospect Theory, while the dynamic context model follows the competitive processing hypothesis. As we describe in detail below, both models assume that the probability of accepting the gamble is a sigmoid function of the expected utility difference between the gamble and the sure payoff, but differ in the utility functions.

The static sensitivity model assumes that all values are processed with the same valuation function, and loss aversion emerges from the asymmetric valuation function, described with the loss aversion parameter λ and the risk aversion parameter ρ as in the Prospect Theory (Kahneman & Tversky, 1979):

$$\begin{array}{c} u\left(x\right)=\{\begin{array}{c} \;{\left|x\right|}^{\rho }\;ifx\geq 0,\\ -\lambda {\left|x\right|}^{\rho }\;ifx<0. \end{array} \end{array}$$ (1)

The dynamic context model assumes that the valuation function does not depend on whether a value is gain or loss, but on how much attention is paid to the value (Yechiam & Hochman, 2013b). The gain and loss in the gamble may have different valuation functions because they need to compete for attention with each other and are not necessarily equally competitive. In contrast, whether a sure payoff is a gain or loss does not influence the attention it receives because it is presented separately at the beginning of each trial. Therefore, we used linear utility functions with different slopes for the three different types of values the participant could pay attention to, that is, sure payoff, gain in the gamble, and loss in the gamble:

$$\begin{array}{c} u\left(x\right)=\{\begin{array}{c} u_{s}x\;\;\;\;\;\text{for}\;\text{sure}\;\text{payoff},\\ u_{g}x\;\;\;\text{for}\;\text{gain}\;\text{in}\;\text{gamble},\\ u_{l}x\;\;\;\;\text{for}\;\text{loss}\;\text{in}\;\text{gamble}. \end{array} \end{array}$$ (2)

Following previous gambling studies that used a single probability of 0.5 (Canessa et al., 2013; Tom et al., 2007), we omitted any possible probability distortions and computed the expected utility of the gamble for both models as:

$$\begin{array}{c} EU_{gamble}=\sum_{i}\;p_{i}u\left(x_{i}\right)=0.5u\left(x_{gain}\right)+0.5u\left(x_{loss}\right) \end{array}$$ (3)

The probability for the participant to accept the gamble is a softmax function of the difference between the expected utility of the gamble and the utility of the sure payoff:

$$\begin{array}{c} \mathrm{Pr}\left(gamble\right)=\frac{1}{1+\exp\left(-\tau \cdot \left[EU_{gamble}-u\left(x_{sure}\right)\right]\right)} \end{array}$$ (4)

For the static sensitivity model, the inverse temperature τ reflects behavioral consistency. Higher τ indicates a higher tendency to select the option with larger utility, instead of choosing randomly. For the dynamic context model, because τ would be redundant when $u_{s}$, $u_{g}$, and $u_{l}$ can all be freely varied, τ is fixed to 1. As the result, both models have 3 free parameters.

We fit the models to participants’ choices using hierarchical Bayesian modeling. In fitting the models, to facilitate the setting of prior parameters, we rescaled the values in the gambles and sure payoffs by dividing them by 10, to make the values comparable to those used in previous studies that used a similar hierarchical Bayesian estimation (Pornpattananangkul et al., 2019; Sokol-Hessner et al., 2016). For the dynamic context model, the 3 parameters $u_{s},u_{g},u_{l}$ of each participant were assumed to be drawn from group-level distributions defined by 3 different sets of μ and σ. For the static sensitivity model, we used the priors from Pornpattanangkul et al. (2019) shown below. For the dynamic context model, we followed the same prior (Gelman, 2006), and bounded the weights from 0 to 5 using an inverse-probit transformation:

$$\begin{array}{c} \mu_{i}\sim \text{Normal}\left(0,\;1\right)\;\text{for}\;i\in \left\{s,g,l\right\} \end{array}$$ (5)

$$\begin{array}{c} \sigma_{i}\sim \text{Cauchy}\left(0,\;5\right)\;\text{for}\;i\in \left\{s,g,l\right\} \end{array}$$ (6)

$$\begin{array}{c} u_{i}^{\prime }\sim \text{Normal}\left(\mu_{i},\sigma_{i}\right)\;\text{for}\;i\in \left\{s,g,l\right\} \end{array}$$ (7)

$$\begin{array}{c} u_{i}=5\cdot \Phi \left(u_{i}^{\prime }\right)\;\text{for}\;i\in \left\{s,g,l\right\} \end{array}$$ (8)

Here, Φ(⋅) is the cumulative distribution function of a standard normal distribution.

Model fitting was implemented using Hamiltonian Monte Carlo in pymc3 (Salvatier et al., 2016) and python3. For each model, 2000 samples were drawn after a burn-in of 1000 samples on each of 4 converged chains. In the later analysis, we used the mean parameters of each participant to calculate participants’ loss aversion indicators.

Both the static sensitivity and dynamic context models provide measures for individual participants’ extent of loss aversion, which we respectively denote by $\lambda_{S}$ (static model) and $\lambda_{D}$ (dynamic model). For the static sensitivity model, $\lambda_{S}\;$ is simply λ, the loss aversion parameter defined in Eq. 1. For the dynamic context model, we defined loss aversion as the ratio between the loss weight against the gain weight in a gamble $\lambda_{D}=u_{l}\;/\;u_{g}$ (Zhao & Walasek, 2020).

We used the deviance information criteria (DIC, Spiegelhalter et al., 2002) to compare the goodness-of-fit of the models:

$$\begin{array}{c} \text{DIC}=p_{D}+\bar{D\left(\theta \right)},\; \end{array}$$ (9)

$$\begin{array}{c} \text{where}\;p_{D}=\bar{D\left(\theta \right)}-D\left(\bar{\theta }\right)\;\text{and}\;D\left(\theta \right)=-2\log\left(p\left(\text{data|}\theta \right)\right) \end{array}$$ (10)

To quantify the performance of models on the individual level, we use the mean parameters of each participant to estimate the response using the two models for each trial. Prediction accuracy was defined as the probability to predict correctly across trials.

#### 2.3.2 MEG acquisition and preprocessing

Participants’ brain activity was recorded by a 306-channel whole-head MEG system. Head position was measured before each block by an isotrack polhemus system with four head position indicator coils (two on the left and right mastoid, the other two on the left and right forehead below the hairline). Horizontal and vertical Electro-oculograms were recorded to monitor eye movement artifacts. Sampling rate was set to be 1000 Hz and an analog band-pass filter from 0.1 to 330 Hz was applied. Maxwell filtering was used to minimize external magnetic interference and to compensate for head movements.

Standard preprocessing procedures were applied using Python3 and the MNE package (Gramfort, 2013). For each participant, the MEG data of all 10 blocks were first concatenated together, and then filtered below 40 Hz. Independent component analysis (ICA) with 40 components was applied to remove artifacts, including blinks, eye movement, heart activity, and low-frequency trends. The resulting signals were down-sampled to 128 Hz and normalized across time. The data sequence was subsequently segregated into trials. Only regular trials, that is, those that had 7 gambles, entered later analysis. Two participants had one invalid trial because of recording errors during the experiment. These two trials were excluded. We then divided the data sequence into 3000 ms segments between 500 ms before and 2500 ms after the onset of each gamble in normal trials. There were on average 1084 segments (ranging from 1064 to 1106 segments) for each participant, thus approximately 136 segments (ranging from 133 to 138 segments) in each gain or loss level.

#### 2.3.3 Time-resolved representational similarity analysis using time-frequency series

To integrate magnetometer and gradiometer signals, we first normalized each channel across time for each participant. The normalized signal was then decomposed into time-frequency powers at 20 frequencies for each channel, using the multitaper method (MNE function *tfr_multitaper*). The frequencies were linearly spaced between 1 to 20 Hz, because they included most of the neural oscillations relevant to value representation (Hunt et al., 2012; Pornpattananangkul et al., 2019). Alpha band (8–12 Hz), which was assumed to be negatively correlated to attention level (Foxe & Snyder, 2011), was also included.

We then averaged the segments of MEG time-frequency series, that is, powers between 500 ms before and 2500 ms after the gamble onset, across trials of the same value for gamble gain or loss, which produced 16 time series for eight gain values and the other eight loss values. Separately for gain and loss, we then calculated the Euclidean distance between each pair of values, such as +10 and +30, resulting in an 8 × 8 representational dissimilarity matrix (RDM) at each time point. To quantify how accurate the values at each time point were encoded, we measured the Kendall’s τ correlation between the neural RDM and the model RDM in their upper triangles (diagonals excluded), following the procedure of Luyckx et al. (2019).

#### 2.3.4 Cluster-based permutation tests of encoding strength

Cluster-based permutation analysis (Maris & Oostenveld, 2007) was used to test whether the encoding strength at a specific time frame was significantly higher than the chance level.

We first estimated the clusters in the RDM correlation time series on the group level, either for all participants or groups divided according to their behavioral loss aversion. The *t*-statistics across participants were used to define clusters, with any consecutive time points with uncorrected *p* < 0.05 defined as one cluster, whose size is the summation of *t*-statistics in the cluster.

We used permutation tests to determine whether a specific cluster found above was significantly larger than that out of chance, that is, when there is no correlation between the neural and model RDMs. We generated the baseline RDM correlation time series by shuffling columns or rows of the model RDM. This shuffling was equivalent to the shuffling of the columns and rows of neural RDM when calculating the correlation between them. That is, for each permutation sample, we used a random model RDM, calculating clusters and cluster sizes as described above. We repeated this process for 1000 times to generate 1000 permutation samples for the distribution of the largest cluster sizes. This distribution was used as the baseline to test the significance of clusters in the real data. When compared to the baseline, the *p* value of a specific real-data cluster was calculated as the probability that a sample cluster was larger than the cluster. Compared to the independent shuffling of the value labels of different trials (Luyckx et al., 2019), which tests the null hypothesis that there is no neural representation at all, our permutation test was stricter, testing the null hypothesis that the neural representation, if any, does not follow the order of value magnitude. In other words, only when there is a neural representation for value (gain or loss), can the null hypothesis be rejected by our permutation test.

We divided all participants evenly into two groups according to their λ estimated in the dynamic context model and further investigated the difference between the high- and low-λ groups (i.e., higher and lower λ). To test the significance of the difference, we shuffled the grouping of participants instead of the columns or rows of RDMs. In each permutation, the participants were evenly but randomly divided into the pseudo high- and low-λ groups. Clusters were calculated based on the *t*-statistics of group difference. As before, the permutation was repeated 1000 times and the *p* value of a real-data cluster was defined as the probability that a permutation cluster was larger than the real-data cluster.

#### 2.3.5 Permutation tests of temporal statistics

To test whether there was any difference between the high- and low-λ groups in the time course of gain and loss valuation, we applied the following permutation test to clusters with significant correlation, obtained from the cluster-based permutation test above. It turned out that there was only one significant cluster for each group and each condition to test.

We tested against the null hypothesis that the temporal statistics (start time, end time, middle time, or duration) of the clusters were the same across different groups. For each time-evolving representation, the first (last) time point in the only significant cluster, whichever exists, was defined as the start (end) time. The middle time was defined as the mean of the start and end times. To further test the length of the representation, we also calculated the duration from the start time to the end time. To generate the null distribution for a specific temporal statistic, we randomly permutated the group labels of different participants and calculated the temporal statistics of the clusters in the spurious groups using the same procedure as for the real data. For those with more than one cluster, the statistics of the largest cluster were used. For those with no significant clusters, all their temporal statistics were set to 0. Such permutation (resampling) was performed for 1000 times to obtain the null distribution of group differences. We then compared the real group difference with the null distribution, calculating the significance level as the percentage of spurious samples higher or lower than the real data.

We performed a similar test for the same set of measures between the gain and loss clusters in each group. For this purpose, we did not shuffle the groups’ members, but shuffled the gain-loss label within each participant.

## 3 Results

Participants completed a decision task while their brain activities were recorded by MEG. On each trial (Fig. 1A), participants first saw a sure payoff and then a length-varying sequence of gambles, each with half chance of monetary gain and loss. Their task was to decide whether to accept the sure payoff or the last gamble. Because participants could not know when the sequence would end, they needed to pay attention to each gamble. The sure payoff, the gain and the loss in the gamble ranged respectively from −20 to +20, from +10 to +80, and from −10 to −80, with three lines of values randomly and independently selected.

We first visualized the choices aggregated over all participants (*n* = 26). Similar to previous studies, participants' choices of accepting the gamble increased with its expected value and decreased with the sure payoff (Fig. 1B). As a group, participants exhibited the tendency of loss aversion—overweighting the potential loss compared to gain, with the probability of accepting gamble being lower than 50% when gain and loss were balanced (Fig. 1C). These choice patterns showed little practice or fatigue effects, according to a comparison of the first and second halves of the experiment (Fig. S10).

### 3.1 Behavioral modeling: evidence for the competitive processing of gain and loss

We constructed a static sensitivity model and a dynamic context model (Fig. 2A and 2B). The static sensitivity model was based on the Prospect Theory, whose utility function for losses is assumed to be λ times of that for gains, no matter whether the gains or losses are in the gamble or sure payoff. In contrast, the dynamic context model assumes different utility functions for the sure payoff ($u_{s}$), the gain in the gamble ($u_{g}$) and the loss in the gamble ($u_{l}$), according to the amount of attention these magnitudes might receive. That is, it assumes a common utility function for gain and loss when they serve as the sure payoff, but different utility functions when they are in the gamble, competing for attention.

We fit the two models (both with 3 free parameters) separately to participants’ choice data using hierarchical Bayesian estimation (see Methods and Fig. S1). According to the deviance information criteria (DIC, Spiegelhalter et al., 2002), the dynamic context model was superior to the static sensitivity model by a likelihood ratio of $4.03\times 10^{14}$:1 (DIC difference: 65.70). We also visualized the predictive accuracy of each model for individual participants’ choices (Fig. 2C, see Methods), which shows that the dynamic context model had better predictions in 19 and equal predictions in 4 out of 26 participants.

Given that the static sensitivity model used power utility functions while the dynamic context model used linear utility functions, one may wonder that the better predictive power of the latter might come from the linear utility function. To exclude this possibility, we also constructed a dynamic context model with power utility functions, which again outperformed the dynamic context model (DIC difference: 75.37, see Fig. S4).

Both the static sensitivity and dynamic context models can be used to quantify loss aversion—the overweighting of losses compared to gains. For the static sensitivity model, λ is the conventional index for loss aversion, which we denote by $\lambda_{V}$ here. For the dynamic context model, the index of loss aversion is defined as the ratio between the loss weight against the gain weight in gambles, that is, $\lambda_{D}=u_{l}\;/\;u_{g}$ (Zhao et al., 2020). A greater-than-one $\lambda_{S}$ or $\lambda_{D}$ would indicate loss aversion. We found that the estimated $\lambda_{S}$ or $\lambda_{D}$ were highly correlated (Pearson’s *r* = 0.653, *p* < 0.001). For both models, the mean λ across all participants was greater than one, though it was marginally significant for the dynamic context model ($\bar{\lambda_{S}}=1.733,\;CI_{95\%}=\left[1.187,\;2.376\right],\;p=0.006$; $\bar{\lambda_{D}}=1.359,\;CI_{95\%}=\left[0.994,\;1.818\right],\;p=0.054$). This finding of loss aversion is consistent with previous studies (Walasek et al., 2018) as well as our visualizations in Figure 1C. We will use the parameter of the winning model (the dynamic context model), $\lambda_{D}$, for further analysis.

### 3.2 Time-resolved encoding strength of neural valuation for gain and loss

We used the representational similarity analysis (RSA, Carlson et al., 2013; Kriegeskorte, 2008) to obtain a time-resolved encoding strength of the valuation of gain and loss from the MEG time series. The basic idea is that similar values induce similar neural activities (e.g., 10 and 20 would induce more similar neural activities than 10 and 80). Because the gain and loss in each gamble were statistically independent of each other, we could separately show the encoding strength of the simultaneously presented gain and loss. Following Luyckx et al. (2019), we constructed the model representational dissimilarity matrix (RDM) as numerical distances between the values, separately for gain and loss values. For each participant and each time point, we computed neural RDM based on the 1–20 Hz time-frequency transform of all channels (see Methods) and calculated the Kendall’s correlation between the model and the neural RDMs.

**Fig. 3.**
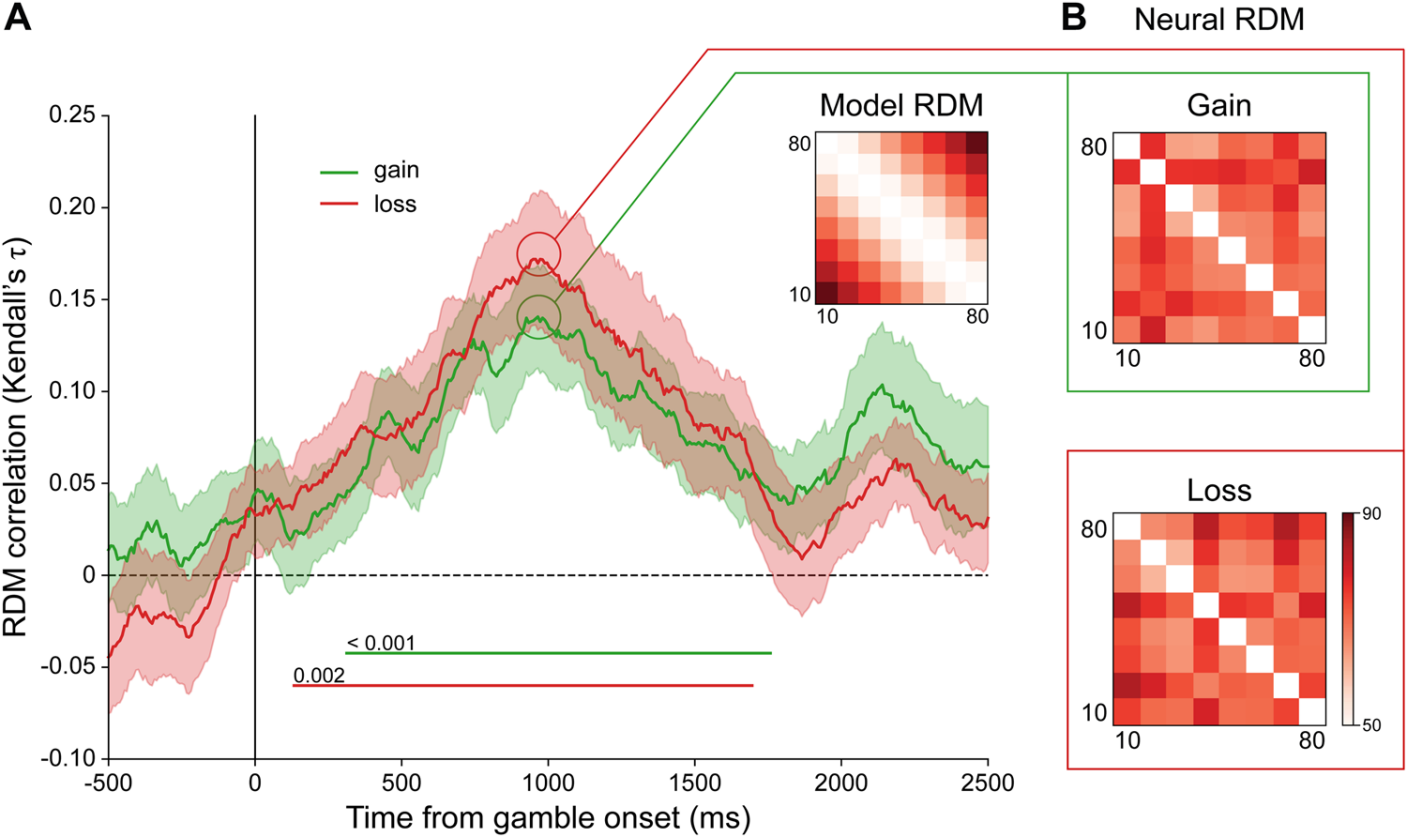
Time-resolved RSA results for gain and loss. (A) The correlations (Kendall’s τ) between the model RDM and the RDMs of gain (green curve) and loss (red curve) over time. Shade denotes standard error. Clusters significantly above the chance level (*p* < 0.05 by permutation test) are indicated by horizontal lines (with the exact *p* value), separately for gain and loss. (B) The neural RDMs for gain and loss at the highest peaks of the correlation curves in (A).

The time-resolved encoding strength (i.e., RDM correlation), averaged over all participants, is shown in Figure 3. According to cluster-based permutation tests, we found above-chance valuation of gain during 313–1758 ms and of loss during 133–1695 ms after the onset of each gamble. Though losses seemed to be represented earlier and longer than gains, the differences did not reach statistical significance.

To exclude the possible contamination of response-related neural signals, we also performed a control analysis that used the same RSA procedure but excluded the last gamble of each trial. The resulting time courses of valuation (Fig. S5) were almost the same as observed here.

### 3.3 Late-stage stronger neural valuation of loss in more loss-averse individuals

**Fig. 4.**
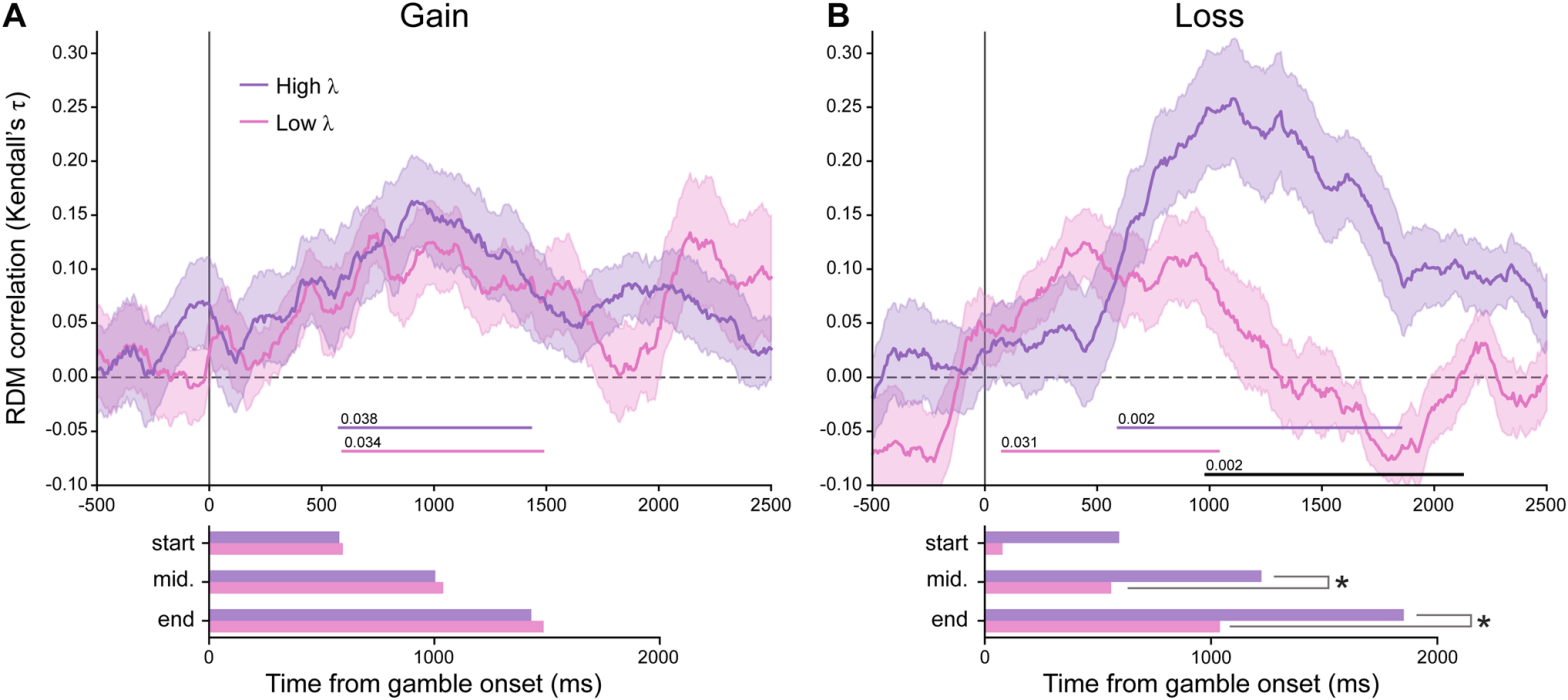
The time-resolved representational similarity results (i.e., Kendall’s τ correlation between the model and neural RDMs) for gain versus loss, and the high (λ=3.06) versus low (λ=0.96) λ groups. (A) The two groups had similar valuation of gain. (B) Compared with the low-λ (lower loss-averse) group, the high-λ (higher loss-averse) group had stronger neural valuation of loss during 984–2125 ms. Shadings denote standard error across participants. Colored and black horizontal lines respectively denote significant clusters on each time course and the difference between the two (*p* < 0.05 by permutation test), with the exact *p* values marked above the lines. The bar plot on the right of each time course plot contrasts the two groups in the start time, end time, middle time, and the duration of its significant cluster. *p < 0.05 by permutation test.

To understand how the neural valuation of gain and loss may relate to the behavioral loss aversion, we divided the 26 participants evenly into two groups with higher and lower loss aversion according to their estimated $\lambda_{D}$ and compared the time course of their neural valuation (Fig. 4). Precisely speaking, only the high-λ group (mean 3.06, CI = [2.04, 5.83]) was loss-averse, while the low-λ group (mean 0.96, CI = [0.93, 0.99]) was even slightly gain-seeking.

The two groups of participants had similar neural valuation of gain over time (Fig. 4A). However, the high-λ group had a stronger valuation of loss than the low-λ group between 984 ms to 2125 ms after gamble onset (Fig. 4B). Note that each gamble was presented for only 1000 ms and the next gamble was presented starting from 1250 ms. That is, individuals who were more loss averse had a stronger neural valuation of the loss during a late stage far beyond the visual presentation of the gamble.

We performed several control analyses to confirm this group difference in loss valuation. First, to exclude the possibility that this difference arose from the response-related neural signals of the last gamble instead of the valuation process, we performed a control analysis where the MEG signals from the last gamble were not included, which led to similar results (Fig. S5). Second, we compared the low- and high-λ groups based on the $\lambda_{S}$ of the static sensitivity model and the $\lambda_{D}$ of the dynamic context model, which were exactly the same. Third, we reached similar conclusions for high-λ individuals when using correlation analysis instead of dividing participants into two groups. In particular, we calculated the area under each RDM correlation curve for each participant during the 500–2000 ms time window and found that the area under the loss curve was positively correlated with the participant’s $\lambda_{D}$ (Spearman’s *r* = 0.54, *p* = 0.004, Fig. S2).

We further examined whether the latency or duration to encode loss was different between the two groups. We used the start, end, and middle times as well as the duration of the time range with significant encoding strength to characterize the time course of valuation (see the bar graphs in Fig. 4). We found that compared to that of the low-λ group, the valuation interval of the high-λ group was 813 ms delayed in the end point and 664 ms delayed in the middle point (permutation test, *p* = 0.015 and *p* = 0.017 respectively). Other differences failed to reach statistical significance (see also Fig. S3). That is, the more loss-averse individuals ended their processing of loss later than the less loss-averse individuals.

## 4 DISCUSSION

We combined computational modeling and MEG to investigate the neural dynamics of gain and loss valuation in a gambling task. In modeling participants’ behavioral choices, we found that the utility functions for gain and loss depend on whether they belong to gambles or sure payoffs, which challenges the conventional view of loss aversion and which is more consistent with a dynamic context valuation process where simultaneously presented gain and loss may compete with each other for attention resource. Brain activities recorded by MEG during the presentation of the gambles allowed us to further characterize the temporal course of competitive neural encoding of gains and losses. We found that participants who had higher loss aversion had a stronger late valuation of loss than those with lower aversion.

One contribution of the present study is to characterize how the valuation of gain and loss unfold across time. Because the temporal resolution of fMRI is only on the scale of seconds (Breiter et al., 2001), existing fMRI studies that find brain activities more sensitive to loss than to gain (Canessa et al., 2013; Tom et al., 2007) cannot tell whether loss is encoded longer than gain or simply stronger than the latter. The temporal dynamics can be better understood by EEG or MEG studies with temporal resolution of milliseconds. However, most previous EEG and MEG studies of loss aversion have a different focus. Some use resting-state alpha oscillation asymmetry to detect individual differences in loss aversion (Duke et al., 2018). Others investigate the neural response to the outcome feedback after decision making (Gehring & Willoughby, 2002), conflict- or response-related ERP (Heeren et al., 2016; Zeng et al., 2019). Still others focus on how the expected utility and variance of the gamble may influence decision making (Pornpattananangkul et al. 2019), overlooking the temporal neural dynamics during valuation. In contrast, by using MEG to record participants’ brain activities when they watched a sequence of gambles without making any decisions, we achieved a clear and time-resolved measure of the valuation process, separately for gain and loss.

Our decoding results were consistent with previous findings that decision-related values are represented starting from 300 ms after the gamble onset (Heeren et al., 2016; Hunt et al., 2012). In addition, we found the representation lasted up to 1500 ms, which was 500 ms after the gamble had disappeared. Similar to previous studies (Hunt et al., 2012; Pornpattananangkul et al., 2019), we found a broadband representation of gains and losses, which included but was not specific to the alpha band activities (Figs. S8 & S9).

In studies that model loss averse decisions as the consequence of evidence accumulation, the differences between loss and gain in evidence accumulating rates are often assumed to be constant across the valuation process (Clay et al., 2017; Sheng et al., 2020; Zhao et al., 2020), except for random fluctuations or the influence of fixations (Krajbich et al., 2012; Sheng et al., 2020). That we found stronger late valuation of loss for more loss-averse individuals suggests that the bias is not constant but inhomogeneous across time and the later valuation may play a more important role than the earlier valuation in human choices.

Theoretically, our computational modeling and MEG results help to distinguish between two alternative hypotheses about the origin of loss aversion: the encoding sensitivity hypothesis and the competitive processing hypothesis. Starting from the Prospect Theory, loss aversion is often considered as a weighting bias, that is, a greater sensitivity to loss compared to gain in both behavioral (Tversky & Kahneman, 1991) and neural (Canessa et al., 2013, 2017) responses. It also proves to be a stable personal trait that is largely irrelevant to tasks (Camerer, 2005; Canessa et al., 2017; Gächter et al., 2022). However, most previous findings are also compatible with an alternative hypothesis that loss aversion is the consequence of an attentional bias towards loss (Yechiam & Hochman, 2013b). This competitive processing hypothesis differs from the conventional view in that it does not assume utility functions are solely valence-based, differing between gain and loss; instead, the utility function of a gain or loss may depend on the context and dynamics of the value processing.

The experimental design of most previous studies (Pornpattananangkul et al., 2019; Tom et al., 2007) made it impossible to differentiate between the dynamic context and static sensitivity hypotheses of loss aversion, because they asked participants to choose between a 50–50 gamble and a sure payoff of 0, where the utilities functions could only be assessed in gambles but not in sure payoffs (Walasek & Stewart, 2021). The dynamic context and static sensitivity models in our study would be reduced to the same one when applied to these studies. To tell apart the two hypotheses, we used a variable sure payoff to replace 0 as the sure option against the gamble. The winning model in fitting, participants’ choices was the dynamic context model (no matter whether its utility function is in a linear or power form), according to which participants’ sensitivity to a value differed for gain and loss only when it needed to compete with simultaneously presented other values.

The neural valuation process revealed by our MEG data is also more consistent with the dynamic context hypothesis in that the differences were specifically in the late stage instead of distributed over the entire time of processing. Decision involving loss has been widely reported to be prolonged (Xue et al., 2009; Yechiam & Telpaz, 2011), and we revealed what occurs in the extended period. Furthermore, we found that participants with lower and higher loss aversion differed in their neural valuation of loss but not in gain, which is consistent with previous fMRI findings that the individual differences in behavioral loss aversion have a stronger correlation with the neural representation of loss than that of gain (Canessa et al., 2013; Tom et al., 2007), especially for emotion-related brain regions such as right posterior insula and parietal operculum (Canessa et al., 2013). It is probably because loss amplifies emotional (Sokol-Hessner et al., 2013) or attentional (Yechiam & Hochman, 2013b) arousal via a “pain matrix,” that is, a network of cortical areas which detects and orients attention towards unpleasant stimuli (Legrain et al., 2011), which is associated with more definitive decisions (Yechiam & Hochman, 2013a) and more sensitive representation of loss (Canessa et al., 2013; Tom et al., 2007).

Our study opens the possibility that temporal dynamics may play an essential role in the neural representations of gain and loss. Future studies may elucidate the potential different roles that different frequency bands play in the asymmetrical representation of gain and loss. Besides, if attention indeed has a causal role in the valuation of gain and loss, we would expect attention manipulation to change the choice behavior of a loss-averse individual to be gain-seeking, or vice versa.

## Supplementary Material

Supplementary Material

## Data Availability

The data and codes are available from the Harvard Dataverse (https://doi.org/10.7910/DVN/GRIKWJ).
