# Supplementary material for: The neural dynamics of loss aversion
Source: Imaging Neurosci (Camb). 2023 Dec 18;1:imag-1-00047. doi: 10.1162/imag_a_00047 (PMC12007549; doi:10.1162/imag_a_00047)
Supplement: Supplementary Material [file imag_a_00047-supp.pdf]

Supplementary figures for

The neural dynamics of loss aversion

Shaozhi Nie, Muzhi Wang, Jian Li, Huan Luo, and Hang Zhang

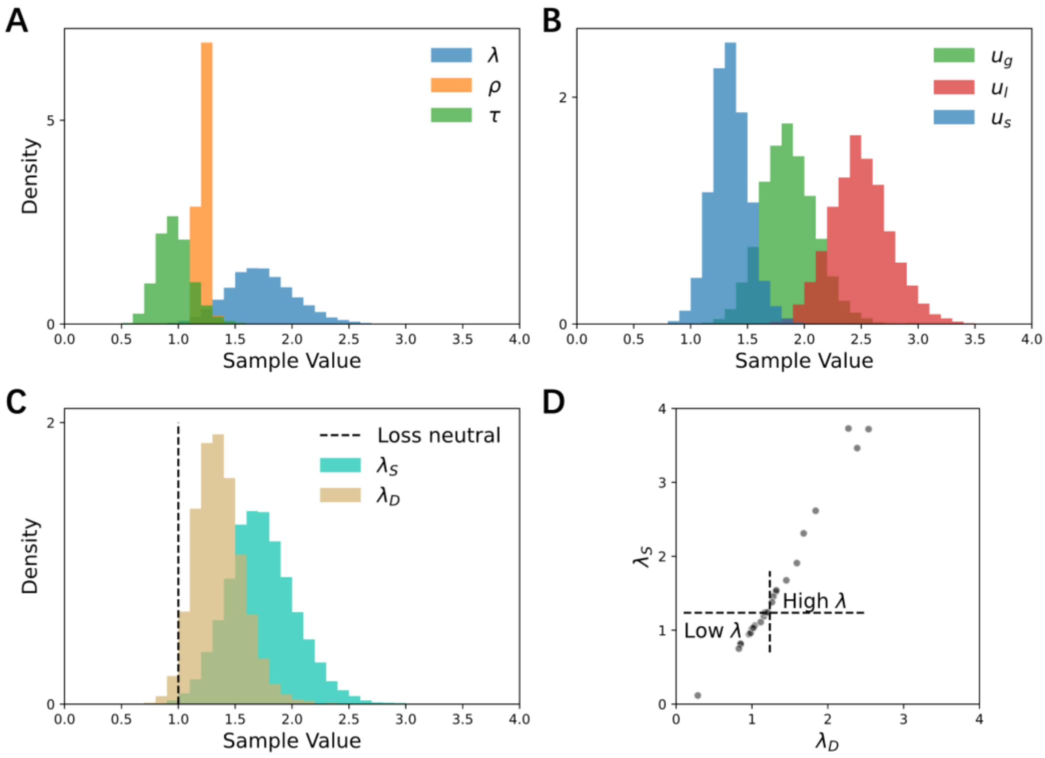

Figure S1. Parameters estimated in two models. (A) Distribution of group-level parameters of the static sensitivity model. Those distributions lay in the similar range as previously reported (Walasek et al., 2018). (B) Distribution of group-level parameters of the static dynamic context model. The weight of gamble loss (u_l) was high than that of gamble gain (u_g). And both of them were higher than the weight of sure payoff (u_s). (C) Distributions of λ_S and λ_D as indicators of loss aversion for the static sensitivity model and the dynamic context model respectively. Most densities of both distributions were great than 1 (loss neutral). (D) Correlation of λ_S and λ_D across participants. Dashed lines indicate criteria grouping participants based on λ_S or λ_D. The grouping was the same regardless of the model used.

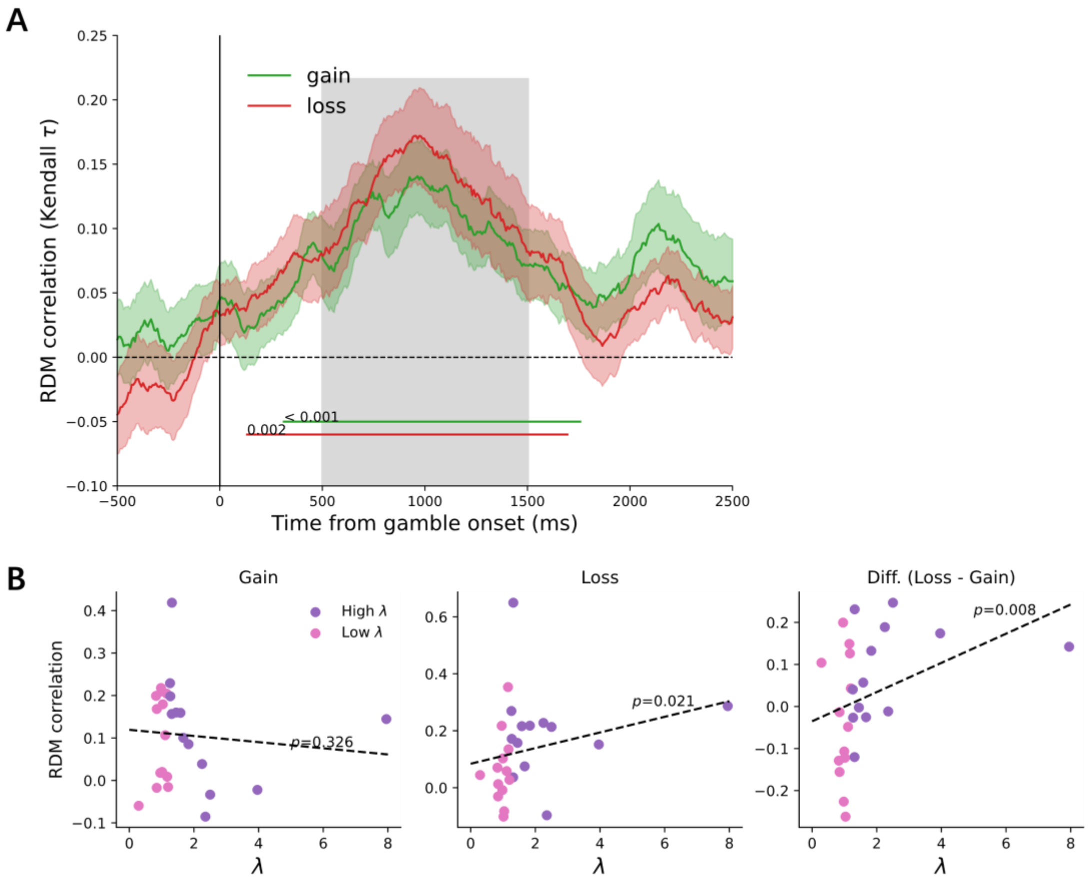

Figure S2. Correlation between RDM correlation and λ across participants. (A) Time window 500-1500ms (gray shade) was selected. For each participant, the averaged RDM correlation in the window was calculated for gain and loss separately as neural indicators of representation. We also calculated the difference in the RDM correlation between the gain and loss. The RDM correlation shown in the figure is the same as article Fig. 3A. (B) Correlation (Spearman's r) between the neural indicators and λ across participants, for gain, loss, and their difference separately. The neural indicators for loss and the difference were significantly positively correlated to λ, but no significance was found for gain.

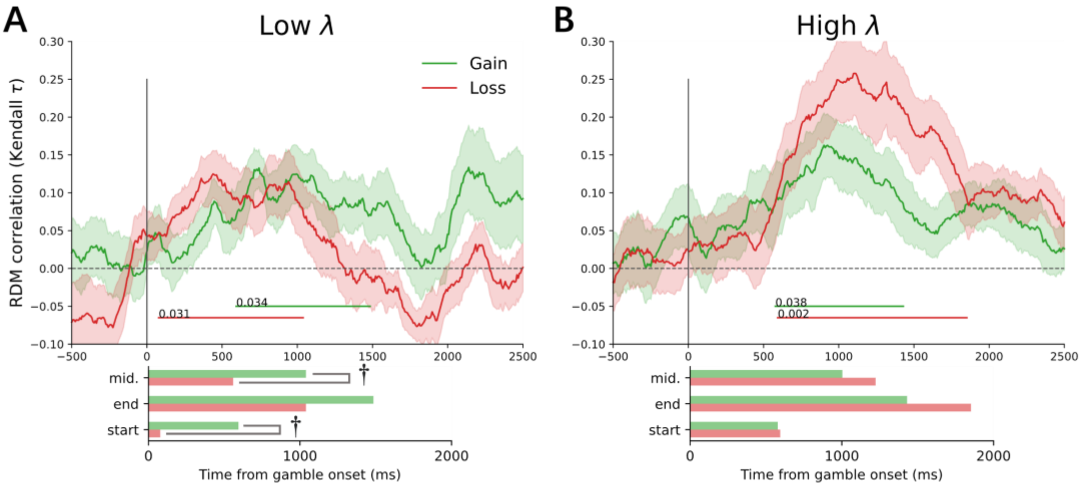

Figure S3. The time-resolved decoding performance (i.e., Kendall's τ correlation between the model and neural RDMs) for gain versus loss, separately for the low-λ group (A) and the high-λ group (B). This is a replot of Figure 4 from a different view. The differences between gain and loss failed to reach significance in either group. Only a marginally significant difference (†: $p < 0.10$) was found for the end and middle time points of the low-λ group (A).

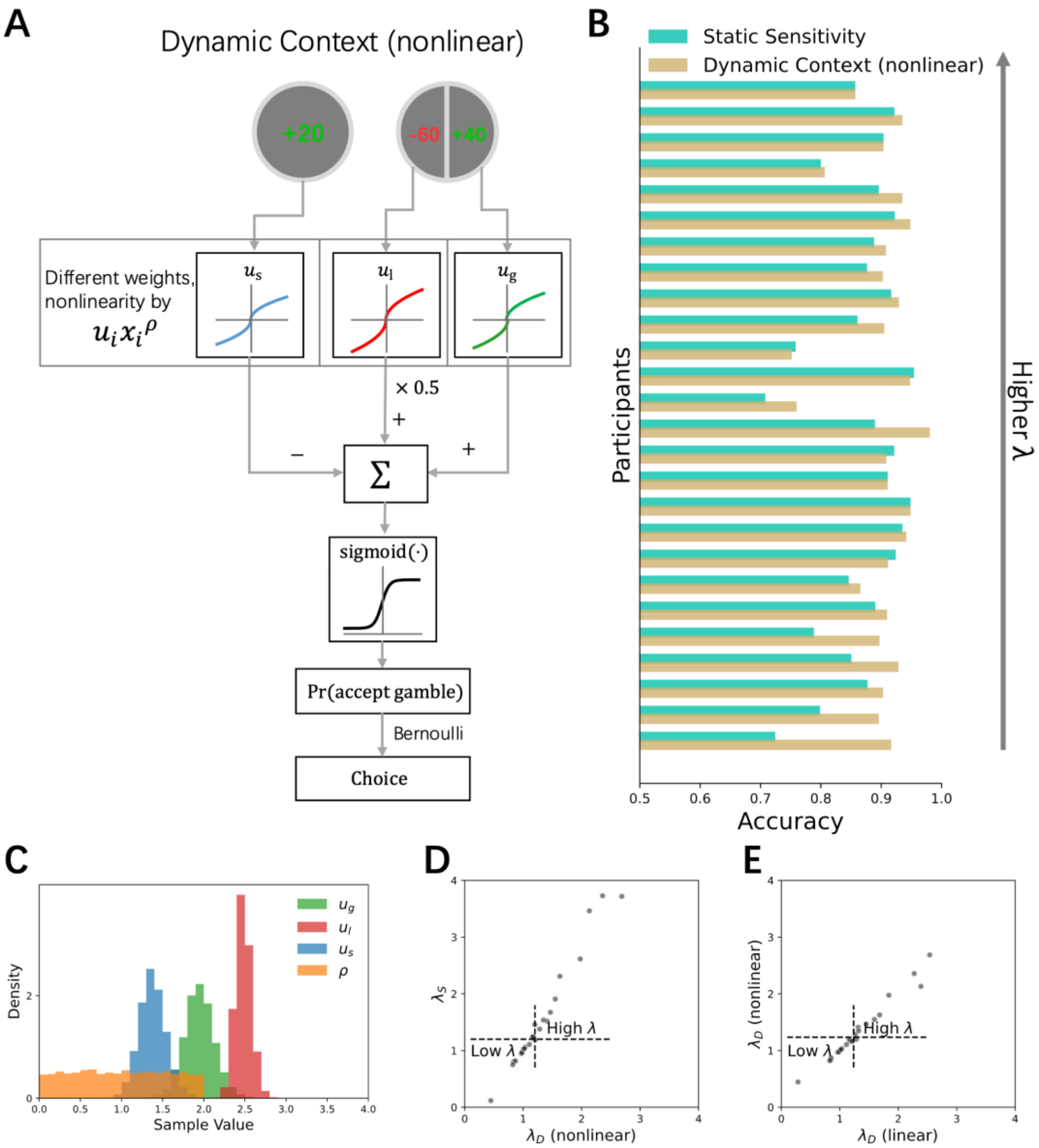

Figure S4. Dynamic context model with nonlinearity, and model comparison. (A) The nonlinear dynamic context model assumes the same nonlinearity but different weights in the utility functions. As well as the static sensitivity model, the nonlinearity was implemented with an exponential parameter ρ. (B) Accuracy of the two models for each participant. We also used DIC to compare the models. The nonlinear dynamic context model outperformed the static sensitivity model (DIC difference: 75.37). (C) Distribution of group-level parameters of the static dynamic context model with nonlinearity. The wide distribution of ρ shows the nonlinearity was redundant. (D) Correlation of λ_S and λ_D (nonlinear) across participants. Dashed lines indicate criteria grouping participants based on λ_S or λ_D. Estimates were highly correlated regardless whether the dynamic context model was linear or not. (E) Correlation of λ_D (linear) and λ_D (nonlinear) across participants, suggesting linearity does not affect the model.

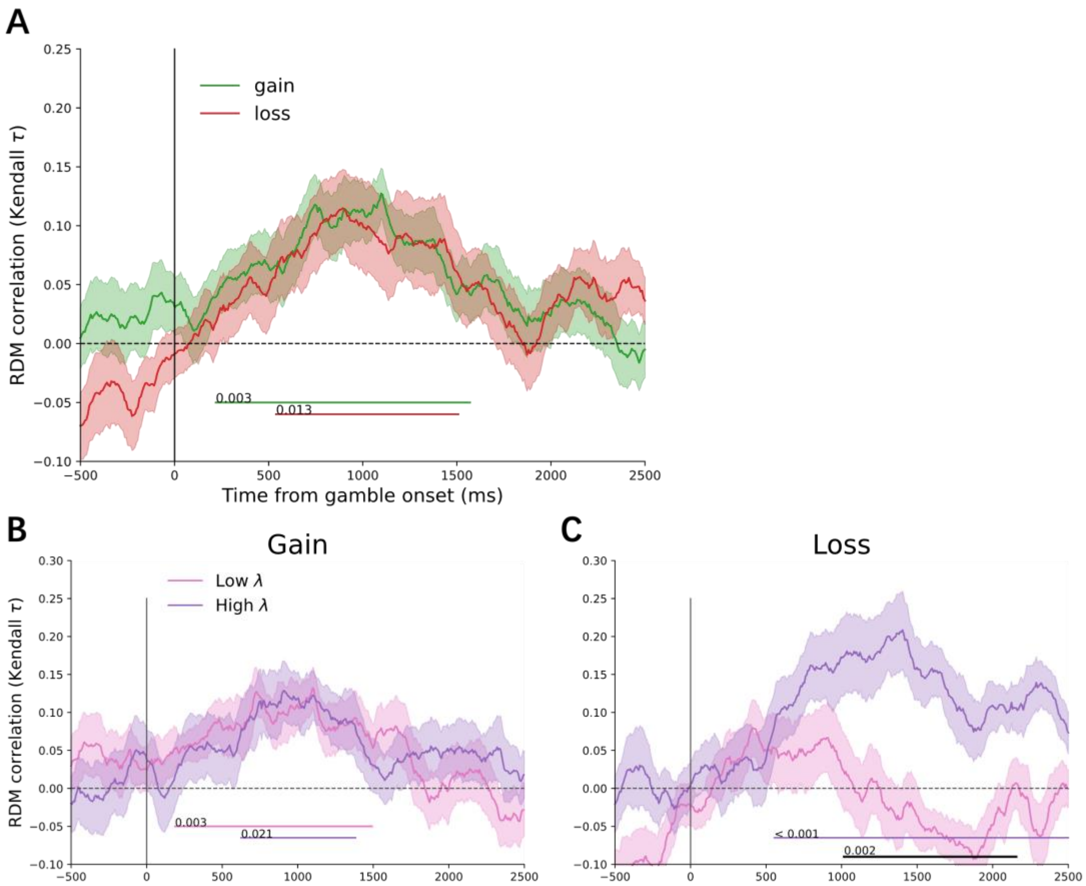

Figure S5. Time-resolved decoding performance (i.e., dissimilarity correlation) for gain and loss, without the last gamble. (A) The correlations (Kendall's τ) between the model RDM and the RDMs of gain (green curve) and loss (red curve) over time. Clusters significantly above the chance level (permutation tests, p < 0.05) are indicated by horizontal lines separately for gain and loss. (B) and (C) The RDM correlations for gain versus loss, and the high ($\lambda = 3.06$) versus low ($\lambda = 0.96$) λ groups.

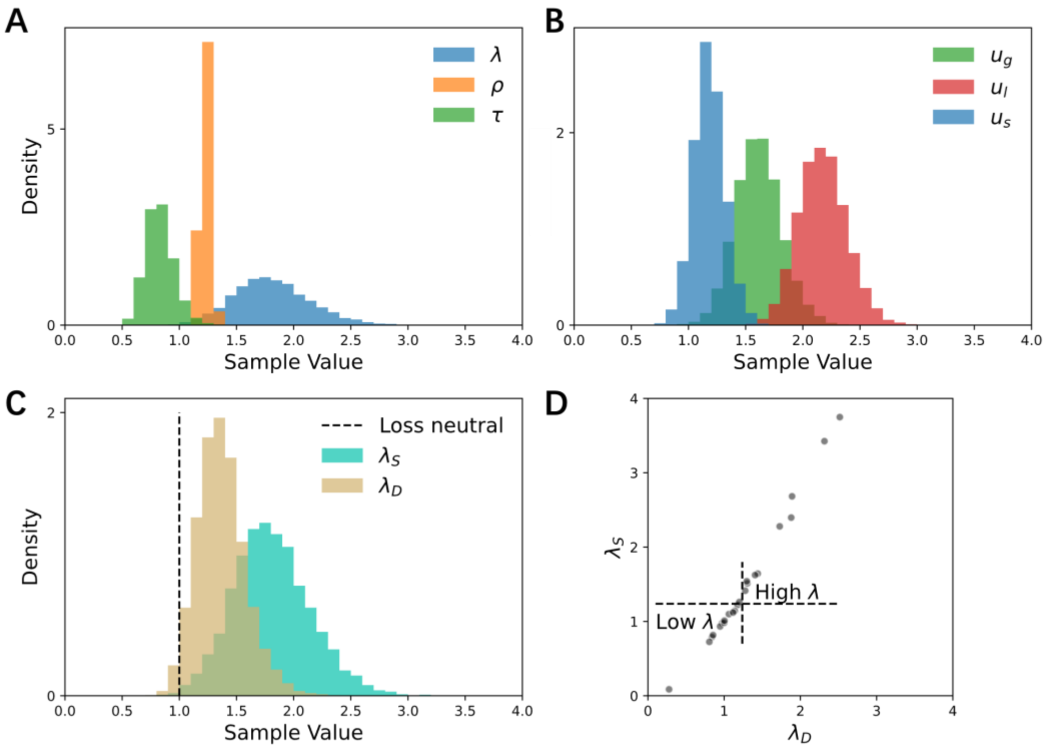

Figure S6. Parameters estimated in two models, including catch trials. (A) Distribution of group-level parameters of the static sensitivity model. (B) Distribution of group-level parameters of the static dynamic context model. The weight of gamble loss (u_l) was high than that of gamble gain (u_g). And both of them were higher than the weight of sure payoff (u_s). (C) Distributions of λ_S and λ_D as indicators of loss aversion for the static sensitivity model and the dynamic context model respectively. Most densities of both distributions were great than 1 (loss neutral). (D) Correlation of λ_S and λ_D across participants. Dashed lines indicate criteria grouping participants based on λ_S or λ_D.

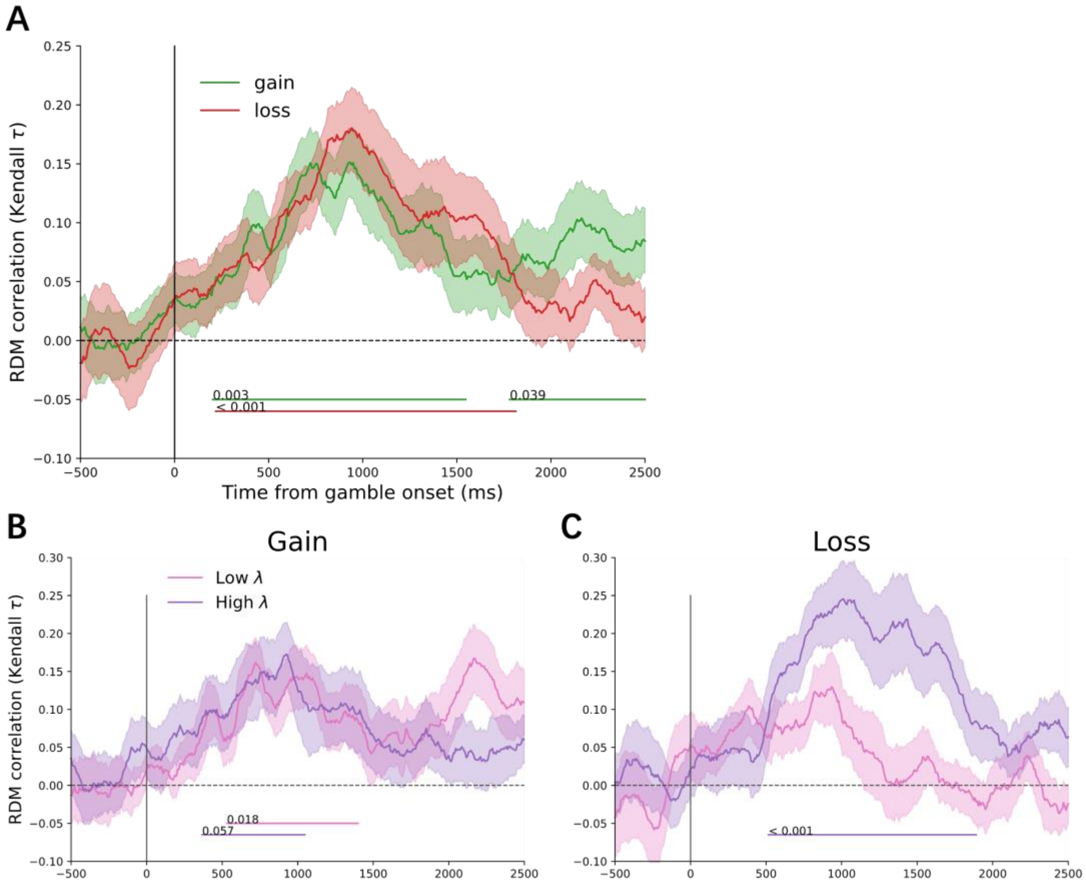

Figure S7. Time-resolved decoding performance (i.e., dissimilarity correlation) for gain and loss, with catch trials. (A) The correlations (Kendall's τ) between the model RDM and the RDMs of gain (green curve) and loss (red curve) over time. Clusters significantly above the chance level (permutation tests, $p < 0.05$) are indicated by horizontal lines separately for gain and loss. (B) and (C) The RDM correlations for gain versus loss, and the high ($\lambda = 3.06$) versus low ($\lambda = 0.96$) λ groups.

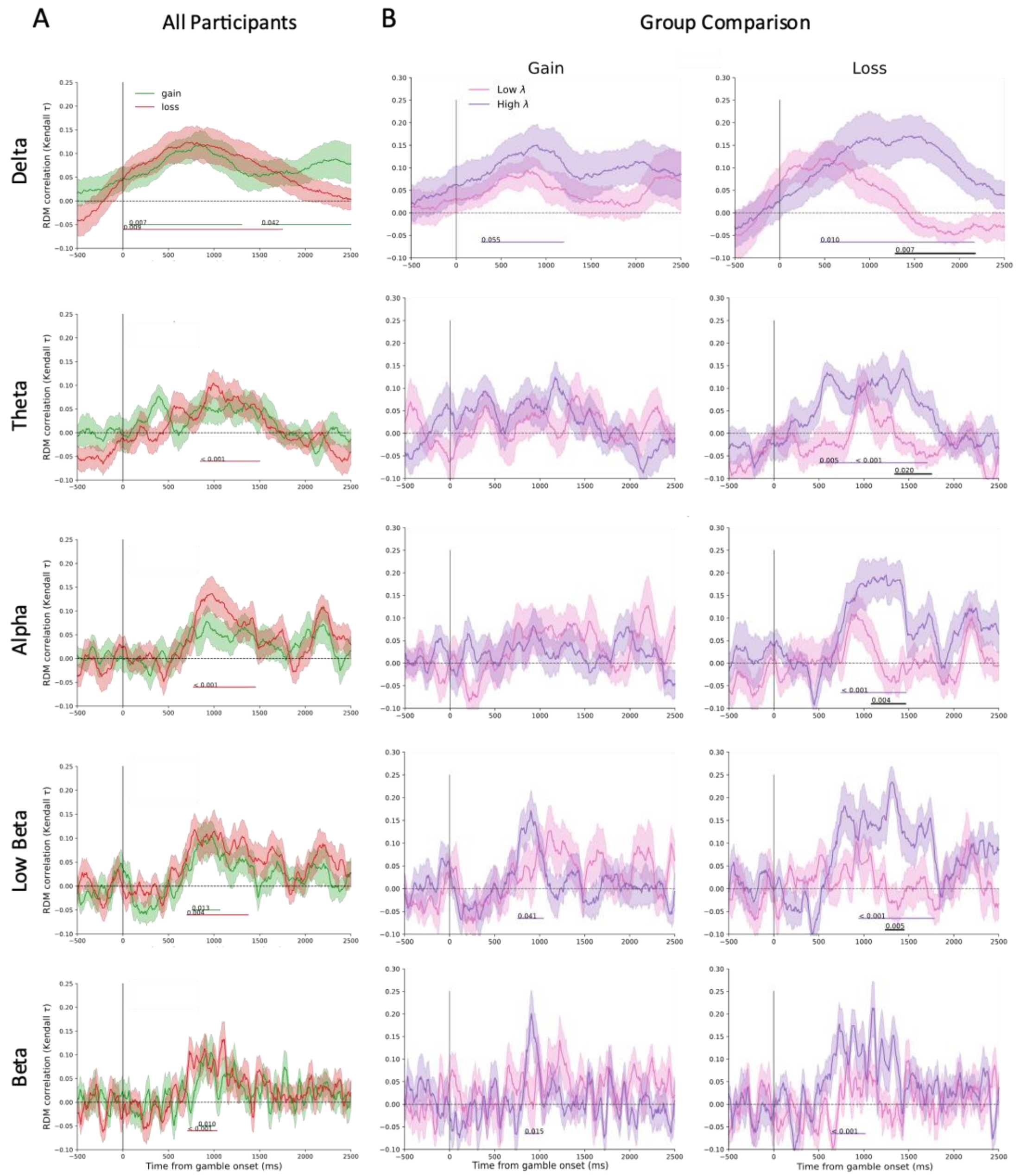

Figure S8. Decoding performance for different frequency bands, following the convention of Fig. 3A and Fig. 4. Left column: The correlations (Kendall's τ) between the model RDM and the RDMs of gain (green curve) and loss (red curve) over time. Clusters significantly above the chance level (permutation tests, $p < 0.05$) are indicated by horizontal lines separately for gain and loss. All frequencies contributed to loss decoding, and three out of the five frequency bands contributed to gain decoding. Middle and right columns: The RDM correlations compared between groups. Colored horizontal lines indicate significant clusters for decoding performance, and black lines indicate significant group difference.

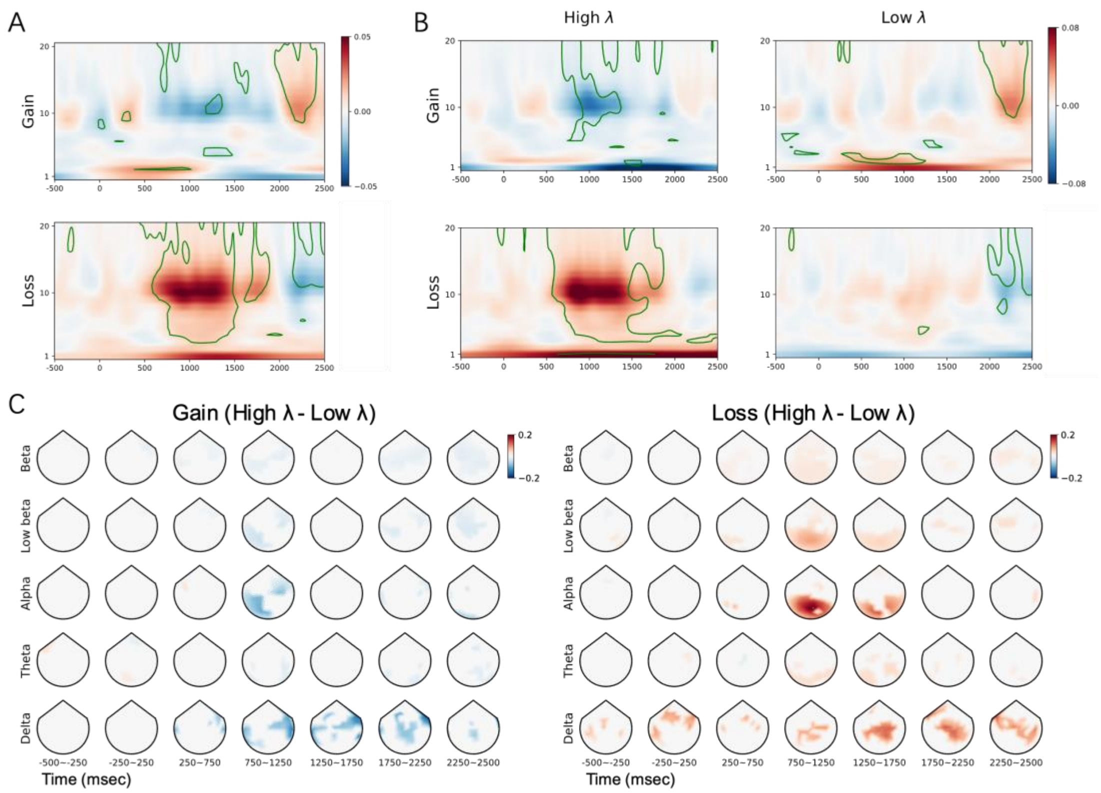

Figure S9. The spectral temporal response functions (STRF) of gains and losses for all and grouped participants, as well as its topography of group difference. STRF was obtained by the coefficients using gamble gains or losses as regressors to predict the amplitudes for frequencies. (A) STRF averaged across all MEG channels. Green contours indicate the significant times and frequencies with uncorrected *t*-test ($p < 0.05$). Though no cluster was tested significant with permutation test, it is observable that alpha band STRF for losses was dominant. (B) STRF for high-λ and low-λ groups separately. We found clusters consistent with the RSA results (Fig. 4) for high-λ group but not high-λ group. (C) Topography for the difference in STRF between groups. The STRF differs mostly in the alpha band in occipito-parietal area and wide-spread delta band activity.

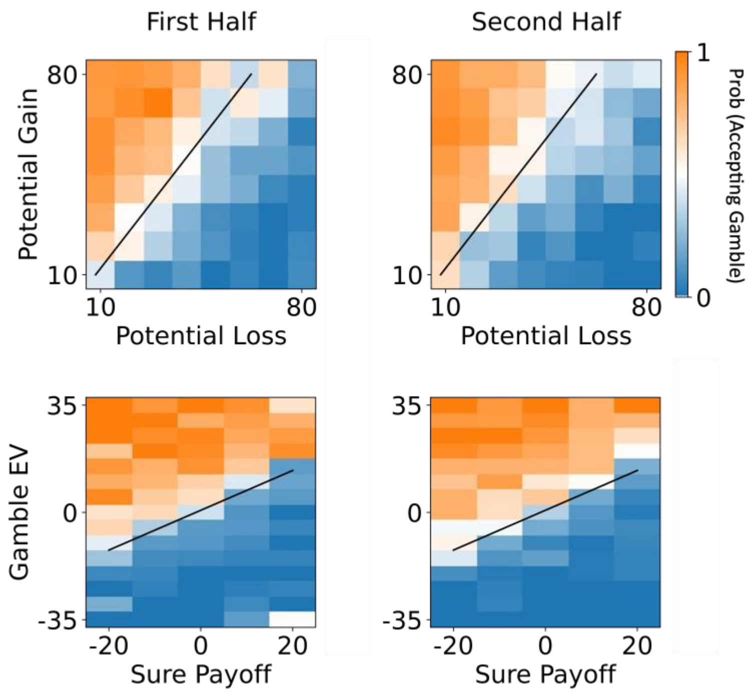

Figure S10. Comparison of participants' decisions between the first and second halves of the 192 trials in the experiment. The top and bottom rows respectively follow the convention of Fig. 1B and 1C. Top row: Probability of accepting the gamble as a function of its expected value (EV) and the sure payoff. Bottom row: Probability of accepting the gamble as a function of its potential gain and loss, averaged across sure payoff levels. The left and right columns are respectively for the first and second halves of the experiment. No cell in the plots was significantly different between the first and second halves (Bonferroni corrected *t*-test, all $p > 0.05$).

Supplementary Reference:

Walasek, L., Mullett, T. L., & Stewart, N. (2018). A meta-analysis of loss aversion in risky contexts. *Available at SSRN 3189088*.
